# A novel hybridity model for TiO_2_-CuO/water hybrid nanofluid flow over a static/moving wedge or corner

**DOI:** 10.1038/s41598-019-52720-6

**Published:** 2019-11-08

**Authors:** Saeed Dinarvand, Mohammadreza Nademi Rostami, Ioan Pop

**Affiliations:** 1 Department of Mechanical Engineering, Islamic Azad University, Central Tehran Branch, Tehran, Iran; 2 Department of Mathematics, Babeş-Bolyai University, 400084 Cluj-Napoca, Romania

**Keywords:** Mechanical engineering, Mechanical engineering, Applied mathematics, Applied mathematics

## Abstract

In this study, we are going to investigate semi-analytically the steady laminar incompressible two-dimensional boundary layer flow of a TiO_2_-CuO/water hybrid nanofluid over a static/moving wedge or corner that is called Falkner-Skan problem. A novel mass-based approach to one-phase hybrid nanofluid model that suggests both first and second nanoparticles as well as base fluid masses as the vital inputs to obtain the effective thermophysical properties of our hybrid nanofluid, has been presented. Other governing parameters are moving wedge/corner parameter (*λ*), Falkner-Skan power law parameter (*m*), shape factor parameter (*n*) and Prandtl number (*Pr*). The governing partial differential equations become dimensionless with help of similarity transformation method, so that we can solve them numerically using bvp4c built-in function by MATLAB. It is worthwhile to notice that, validation results exhibit an excellent agreement with already existing reports. Besides, it is shown that both hydrodynamic and thermal boundary layer thicknesses decrease with the second nanoparticle mass as well as Falkner-Skan power law parameter. Further, we understand our hybrid nanofluid has better thermal performance relative to its mono-nanofluid and base fluid, respectively. Moreover, a comparison between various values of nanoparticle shape factor and their effect on local heat transfer rate is presented. It is proven that the platelet shape of both particles (*n*_1_ = *n*_2_ = 5.7) leads to higher local Nusselt number in comparison with other shapes including sphere, brick and cylinder. Consequently, this algorithm can be applied to analyze the thermal performance of hybrid nanofluids in other different researches.

## Introduction

Nanofluids categorize as solid-liquid mixtures including a carrier medium namely base fluid and nano-size particles. Because of very small dimensions (1–100 nm) and huge specific surface area of the nanoparticles, nanofluids have good thermophysical properties; accordingly, they can be used widely in various aspects of technology consisting of microelectromechanical system (MEMS) and nanotechnologies. It is conventional that the thermal conductivity of nanoparticles, especially, metals, their oxides, graphite, and its derivatives exceeds by several orders the thermal conductivity of traditional working fluids (e.g. water, propylene glycol and different oils). Applying a liquid with dispersed particles as the heat-transfer fluid was started a long time ago; however, the classical scattered liquids cannot apply due to sedimentation of the dispersed particles. Nanofluids do not possess foregoing disadvantages. The first experiments of the nanofluids thermal conductivity have elucidated perfect results: the use of nanoparticles (metals or their oxides) in very small volume fractions significantly enhanced the thermal conductivity of the base fluid. So, these new working fluids may be used in many heat transfer applications, like engine cooling, refrigeration, cooling electronics, solar water heating, thermal storage, and so forth (Aliofkhazraei^1^). Although there are some inconsistencies in the previously published literature and incompliance behaviors of the mechanism of the heat transfer in nanofluids, it has known as an efficient heat transfer fluid. It is worth mentioning that many works on nanofluids can be discovered in the useful books by Das *et al*.^2^, Nield and Bejan^3^, Minkowycz *et al*.^4^, and Shenoy *et al*.^5^, in the review articles by Buongiorno *et al*.^6^, Kakaç and Pramuanjaroenkij^7^, Manca *et al*.^8^, Mahian *et al*.^9^, Sheikholeslami and Ganji^10^, Myers *et al*.^11^, as well as in the research articles by Mehryan *et al*.^12^, Mohebbi *et al*.^13^, Dinarvand *et al*.^14^, Abedini *et al*.^15^, Esfe *et al*.^16^, Nademi Rostami *et al*.^17^, etc. These reviews illustrate in details, the preparation methods of nanofluids, theoretical and empirical findings of thermal conductivity and viscosity of nanofluids, and the mathematical formulation related to convective transport in nanofluids, while research ones study the analytic modeling of single-particle nanofluids or hybrid nanofluids in various complex geometries with the use of single-phase model. This single-phase model is based on Tiwari–Das (see Tiwari and Das^18^) nanofluid model. The foregoing model is valid when there is no relative velocity between the base fluid and the nanoparticles. As a result, we have total thermal equilibrium inside the working fluid including fluid phase and solid phase and therefore, the volume concentration of the nanoparticles will be constant in all points of the bulk fluid. On the other hand, the Tiwari–Das model considers the effective thermophysical properties of the single-phase nanofluid for the dimensional governing partial differential equations, while the only thermophysical property pertaining to similarity variables i.e. kinematic viscosity relates to the base fluid.

Recently, the scientists have also attempted to apply hybrid nanofluids, which are designed by dispersing different nanoparticles either in mixture or composite form (Ranga *et al*.^19^). Applying hybrid nanofluids causes the significant modification in heat transfer and pressure drop specifications by trade-off between advantages and disadvantages of individual suspension, attributed to better aspect ratio, suitable thermal network and especially synergistic influence of nanoparticles. However, the long-term stability, manufacture process, the choice of good nanoparticles combination to get synergistic influence and the expense of nanofluids may be biggest challenges and even beyond the practical applications (Minea^20^). In the last years, the improved heat transfer specifications of the hybrid nanofluids draw the attention of investigators to examine the effect of different nanocomposites in different heat transfer fields, such as heat exchanger (Harandi *et al*.^21^), heat sink (Nimmagada and Venkatasubbaiah^22^), solar collectors (Xuan *et al*.^23^, Rativa and Gómez-Malagón^24^), boiling (Bhosale and Borse^25^), micro power generation (He *et al*.^26^), etc. Madhesh *et al*.^27^ utilized water-based copper–titania hybrid nanofluids, under particle loading ranging from 0.1% to 2% to study the heat transfer characteristics in a shell-and-tube heat exchanger. Besides, the thermal conductivity of the Al_2_O_3_/water, CuO/water and Al_2_O_3_–CuO/water hybrid nanofluid for different temperatures and volume concentrations experimentally investigated by Senthilraja *et al*.^28^. In addition, we mention the review paper by Sarkar *et al*.^29^ and the articles by Ghalambaz *et al*.^30,31^ on hybrid nanoparticles as additives. It should be mentioned here that, the theoretical simulation of single-phase hybrid nanofluids can be applied by the Tiwari–Das model, too. However, we must initially expand the effective thermophysical properties of mono-nanofluids for hybrid nanofluids with two different nanoparticles. This action has been done before by many researchers like Nademi Rostami *et al*.^17^

Falkner–Skan (see Falkner and Skan^32^) similarity boundary-layer problem discusses streaming flows over (static) wedges with arbitrary angle. The Blasius problem is related to the zero angle and the Heimenz problem is related to the ${90}^{\circ }$ angle for a 2D stagnation point (Panton^33^, Tamim *et al*.^34^ and Dinarvand *et al*.^35^). There are so many works in literature remarked as “Falkner-Skan” problem. For example see Cebeci and Keller^36^, Riley and Weidman^37^, Asaithambi^38^, Pantokratoras^39^ and Ishak *et al*.^40^. The steady boundary-layer flow of a non-Newtonian fluid, implemented by a power-law model, over a moving wedge in a moving fluid is investigated by Ishak *et al*.^41^. Yacob *et al*.^42,43^ numerically investigated the steady two-dimensional boundary layer flow over a static/moving wedge immersed in nanofluids with uniform surface temperature and prescribed surface heat flux, respectively. Recently, Nadeem *et al*.^44^ studied the characteristics of induced magnetic field incorporated in a viscous fluid past a static/moving wedge with considering Cu, Al_2_O_3_ and TiO_2_ as the nanoparticles and water as the base fluid. He mentioned that the fluid flow caused by a moving wedge is a remarkable problem in which the fluid and wall velocities are proportional each other, this is useful in the thermal processing of sheet-like substance that is a necessary operation in the paper procurement, wire drawing, drawing of plastic films, polymeric sheets and metal spinning.

According to author’s knowledge, there is no work on the Falkner-Skan problem with considering hybrid nanofluids yet. As a result, we present the problem of boundary layer flow past a static/moving wedge immersed in water-based hybrid nanofluid with constant surface temperature by a mass-based computational algorithm. This algorithm proposes the new definition of an equivalent solid volume fraction, solid density and solid specific heat at constant pressure that are obtained from thermophysical properties of both base fluid and nanoparticles, simultaneously. Then, foregoing parameters along with other relevant governing parameters are substituting into the governing dimensionless ODEs after implementing similarity variables and numerically solved by bvp4c routine. Moreover, the effect of nanoparticles shape factor is considered, too.

## Problem Description and Governing Equations

**Figure 1:**
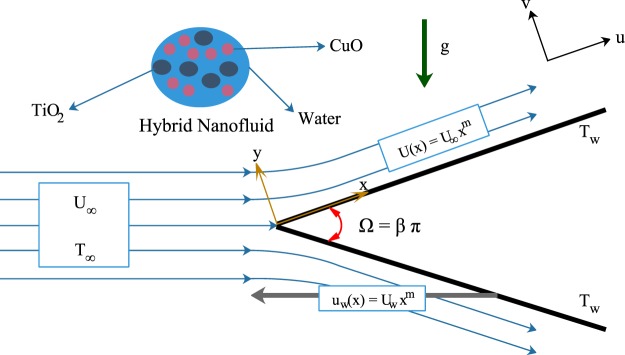
The schematic diagram of the problem and coordinate system.

Assume an incompressible laminar steady two-dimensional boundary layer flow over a static or moving wedge in an aqueous hybrid nanofluid with prescribed external flow and moving wedge velocities as displayed in Fig. 1. We have chosen titania (TiO_2_) and copper oxide (CuO) as nanoparticles with water as base fluid. We also assume that the base fluid and nanoparticles are in thermal equilibrium and no slip occurs between them. It is worth mentioning that, to develop the targeted hybrid nanofluid TiO_2_-CuO/water, titania is initially dispersed into base fluid then, copper oxide is scattered in TiO_2_/water nanofluid. Therefore, the subscript (1) corresponds to first nanoparticle (TiO_2_), while subscript (2) is applied for second nanoparticle (CuO) as well as subscript (f) related to base fluid. Table 1 shows thermophysical properties of the base fluid and the nanoparticles at 25 °C (see Dinarvand and Pop^45^, Nayak *et al*.^46^, Vajjha *et al*.^47^).

**Table 1:** Thermophysical properties of the base fluid and the nanoparticles at 25 °C (Dinarvand and Pop^45^, Nayak *et al*.^46^ and Vajjha *et al*.^47^).

Thermophysical properties	Pure water $({{\bf{H}}}_{{\bf{2}}}{\bf{O}})$	Titania $({\bf{T}}{\bf{i}}{{\bf{O}}}_{{\bf{2}}})$	Copper oxide $({\bf{C}}{\bf{u}}{\bf{O}})$
${C}_{p}({\rm{J}}/{\rm{kg}}\mathrm{.K})$	4179	686.2	533
$\rho ({\rm{kg}}/{{\rm{m}}}^{{\rm{3}}})$	997.1	4250	6500
$k({\rm{W}}/{\rm{m}}.{\rm{K}})$	0.613	8.9538	17.65
Particle size (nm)	—	50	29

According to Fig. 1, we choose 2D Cartesian coordinate system (*x*, *y*) where *x* and *y* are the coordinates measured along the surface of the wedge and normal to it, respectively. It is assumed that the free stream velocity is $U(x)={U}_{\infty }{x}^{m}$ and the temperature of the ambient hybrid nanofluid is ${T}_{\infty }$, while the moving wedge velocity is ${u}_{w}(x)={U}_{w}{x}^{m}$ and its constant temperature surface is ${T}_{w}.$ After using boundary layer approximations and Tiwari-Das nanofluid model (see Tiwari and Das^18^) as well as the Bernoulli’s equation in free stream, the governing non-linear PDEs of mass, momentum and energy can be written as follows (see Yacob *et al*.^42^):

1 $$\frac{\partial u}{\partial x}+\frac{\partial v}{\partial y}=0,$$

2 $$u\frac{\partial u}{\partial x}+v\frac{\partial u}{\partial y}=U\frac{dU}{dx}+\frac{{\mu }_{hnf}}{{\rho }_{hnf}}\frac{{\partial }^{2}u}{\partial {y}^{2}},$$

3 $$u\frac{\partial T}{\partial x}+v\frac{\partial T}{\partial y}={\alpha }_{hnf}\frac{{\partial }^{2}T}{\partial {y}^{2}},$$

subject to the boundary conditions

4 $$\begin{array}{c}u={u}_{w}(x),\,\,v=0,\,\,T={T}_{w}\,at\,y=0,\\ \,\,u\to U(x)\,T\to T\infty \,\,\,as\,y\to \infty .\end{array}$$

In which *u* and *v* are the velocity components along *x* and *y* directions, respectively, *T* is the temperature of the hybrid nanofluid within the thermal boundary layer, *C*_*p*_ is the specific heat at constant pressure, *ρ*_*hnf*_, *μ*_*hnf*_ and *α*_*hnf*_ are the density, the viscosity and the thermal diffusivity of the hybrid nanofluid, respectively, and are defined according to Table 2.

**Table 2:** Applied models for thermophysical properties of the hybrid nanofluid (Sundar *et al*.^48^, Ghadikolaei *et al*.^49^ and Hayat and Nadeem^50^).

Property	Hybrid Nanofluid
Viscosity (*μ*)	$\frac{{\mu }_{f}}{{(1-\varphi )}^{2.5}}$
Density (*ρ*)	$(1-\varphi )({\rho }_{f})+\varphi ({\rho }_{s})$
Heat capacity(*ρC*_*p*_)	$[(1-\varphi )({\rho }_{f})+\varphi ({\rho }_{s})]\times [(1-\varphi ){({C}_{P})}_{f}+\varphi {({C}_{P})}_{s}]$
Thermal conductivity (*k*)	$\frac{{k}_{2}+({n}_{2}-1){k}_{nf}-({n}_{2}-1){\varphi }_{2}({k}_{nf}-{k}_{2})}{{k}_{2}+({n}_{2}-1){k}_{nf}+{\varphi }_{2}({k}_{nf}-{k}_{2})}\times \frac{{k}_{1}+({n}_{1}-1){k}_{f}-({n}_{1}-1){\varphi }_{1}({k}_{f}-{k}_{1})}{{k}_{1}+({n}_{1}-1){k}_{f}+{\varphi }_{1}({k}_{f}-{k}_{1})}\times ({k}_{f})$
Diffusivity (*α*)	$\frac{{k}_{hnf}}{{(\rho {C}_{p})}_{hnf}}$

In Table 2, *k*_*nf*_ is the thermal conductivity of the single nanoparticle’s nanofluid that is computed from Hamilton-Crosser model (see Ghadikolaei *et al*.^49^, and Hayat and Nadeem^50^)

5 $$\frac{{k}_{nf}}{{k}_{f}}=\frac{{k}_{1}+({n}_{1}-1){k}_{f}-({n}_{1}-1){\varphi }_{1}({k}_{f}-{k}_{1})}{{k}_{1}+({n}_{1}-1){k}_{f}+{\varphi }_{1}({k}_{f}-{k}_{1})}$$

where *n* is the empirical shape factor for the nanoparticle and is determined in Table 3,

**Table 3:** The common values of shape factor of nanoparticles (Sheikholeslami and Shamlooei^51^).

Shapes of nanoparticle	n
Spherical	3
Brick	3.7
Cylinder	4.9
Platelet	5.7

Moreover, we propose *ϕ*, *ρ*_*s*_ and (*C*_*p*_)_*s*_ as the equivalent volume fraction for nanoparticles, the equivalent density of nanoparticles and the equivalent specific heat at constant pressure of nanoparticles, respectively, as well as *ϕ*_1_ and *ϕ*_2_ are solid fraction of first and second nanoparticles, respectively, that are calculated from following formulas (see Sundar *et al*.^48,52,53^)

6 $${\rho }_{s}=\frac{({\rho }_{1}\times {w}_{1})+({\rho }_{2}\times {w}_{2})}{{w}_{1}+{w}_{2}},$$

7 $${({C}_{P})}_{s}=\frac{\{{({C}_{P})}_{1}\times {w}_{1}\}+\{{({C}_{P})}_{2}\times {w}_{2}\}}{{w}_{1}+{w}_{2}},$$

8 $${\varphi }_{1}=\frac{\frac{{w}_{1}}{{\rho }_{1}}}{\frac{{w}_{1}}{{\rho }_{1}}+\frac{{w}_{2}}{{\rho }_{2}}+\frac{{w}_{f}}{{\rho }_{f}}},$$

9 $${\varphi }_{2}=\frac{\frac{{w}_{2}}{{\rho }_{2}}}{\frac{{w}_{1}}{{\rho }_{1}}+\frac{{w}_{2}}{{\rho }_{2}}+\frac{{w}_{f}}{{\rho }_{f}}},$$

10 $$\varphi =\frac{\frac{{w}_{1}+{w}_{2}}{{\rho }_{s}}}{\frac{{w}_{1}+{w}_{2}}{{\rho }_{s}}+\frac{{w}_{f}}{{\rho }_{f}}}.$$

we notice that, *w*_1_, *w*_2_ and *w*_f_ are the first nanoparticle, the second nanoparticle and the base fluid masses, respectively.

According to White^54^ we are looking for a similarity solution of Eqs (1–3) along with boundary conditions (4) of the following form:

11 $$\eta ={(\frac{(m+1)U(x)}{2{\nu }_{f}x})}^{1/2}y,\,\psi ={(\frac{2{\nu }_{f}xU(x)}{m+1})}^{1/2}f(\eta ),\,\theta (\eta )=\frac{T-T\infty }{{T}_{w}-T\infty },$$

where $\psi$ is the dimensional stream function and is expressed in the usual form as $u=\partial \psi /\partial y$ and $v=-\partial \psi /\partial x,$ *f* is the dimensionless stream function, *θ* is the dimensionless temperature distribution of the hybrid nanofluid and $\eta$ is independent similarity variable. Fortunately, using similarity transformation method, substituting Eq. (11) into non-linear PDEs (2) and (3) and considering Eqs (8–10), give us a following set of dimensionless non-linear ODEs:

12 $${A}_{1}{f}^{{\prime\prime}^{\prime}}+ff^{\prime\prime} +\frac{2m}{m+1}(1-{ff^{\prime} }^{2})=0,$$

13 $${\frac{1}{Pr}}\,{\frac{{k}_{hnf}}{{k}_{f}}{A}_{2}}\theta ^{\prime\prime} +f\theta ^{\prime} =0,$$

$${A}_{1}={(1-\frac{\frac{{w}_{1}+{w}_{2}}{{\rho }_{s}}}{\frac{{w}_{1}+{w}_{2}}{{\rho }_{s}}+\frac{{w}_{f}}{{\rho }_{f}}})}^{-2.5}{(1-\frac{\frac{{w}_{1}+{w}_{2}}{{\rho }_{s}}}{\frac{{w}_{1}+{w}_{2}}{{\rho }_{s}}+\frac{{w}_{f}}{{\rho }_{f}}}+\frac{\frac{{w}_{1}+{w}_{2}}{{\rho }_{s}}}{\frac{{w}_{1}+{w}_{2}}{{\rho }_{s}}+\frac{{w}_{f}}{{\rho }_{f}}}\frac{{\rho }_{s}}{{\rho }_{f}})}^{-1}$$

$${A}_{2}=\frac{{(\rho {C}_{P})}_{f}}{[(1-\frac{\frac{{w}_{1}+{w}_{2}}{{\rho }_{s}}}{\frac{{w}_{1}+{w}_{2}}{{\rho }_{s}}+\frac{{w}_{f}}{{\rho }_{f}}})({\rho }_{f})+\frac{\frac{{w}_{1}+{w}_{2}}{{\rho }_{s}}}{\frac{{w}_{1}+{w}_{2}}{{\rho }_{s}}+\frac{{w}_{f}}{{\rho }_{f}}}({\rho }_{s})]\times [(1-\frac{\frac{{w}_{1}+{w}_{2}}{{\rho }_{s}}}{\frac{{w}_{1}+{w}_{2}}{{\rho }_{s}}+\frac{{w}_{f}}{{\rho }_{f}}}){({C}_{P})}_{f}+\frac{\frac{{w}_{1}+{w}_{2}}{{\rho }_{s}}}{\frac{{w}_{1}+{w}_{2}}{{\rho }_{s}}+\frac{{w}_{f}}{{\rho }_{f}}}{({C}_{P})}_{s}]}$$

restricted with the boundary conditions

14 $$f(0)=0,\,\,f^{\prime} (0)=\lambda ,\,\,\theta (0)=1,$$

15 $$f^{\prime} (\infty )\to 1,\,\,\theta (\infty )\to 0.$$

Here, the Prandtl number (*Pr*), the constant moving wedge parameter $(\lambda )$ as well as the Hartree pressure gradient parameter (*β*) are defined as

16 $$\lambda =\frac{{U}_{w}}{{U}_{\infty }},\,\beta =\frac{2m}{m+1},\,\,Pr=\frac{{\upsilon }_{f}}{{\alpha }_{f}},$$

It should be mentioned that $\lambda  > {\rm{0}}$ and $\lambda  < {\rm{0}}$ correspond to a moving wedge in same and opposite directions to the free stream, respectively, while $\lambda ={\rm{0}}$ corresponds to a static wedge. Furthermore, $\beta \, > {\rm{0}}$ is caused by negative or favorable pressure gradient, while $\beta  < {\rm{0}}$ creates positive or unfavorable pressure gradient (see White^54^).

The skin friction coefficient *C*_*f*_ and the local Nusselt number *Nu*_*x*_ are defined as

17 $${C}_{f}=\frac{{\tau }_{w}}{{\rho }_{f}{U}^{2}},\,N{u}_{x}=\frac{x{q}_{w}}{{k}_{f}({T}_{w}-{T}_{\infty })}.$$

where, ${\tau }_{w}$ is the shear stress at the surface of the wedge and *q*_*w*_ is the heat flux from the surface of the wedge, which are illustrated by

18 $${\tau }_{w}={\mu }_{hnf}{(\frac{\partial u}{\partial y})}_{y=0},\,\,{q}_{w}=\,-{k}_{hnf}{(\frac{\partial T}{\partial y})}_{y=0}.$$

**Figure 2:**
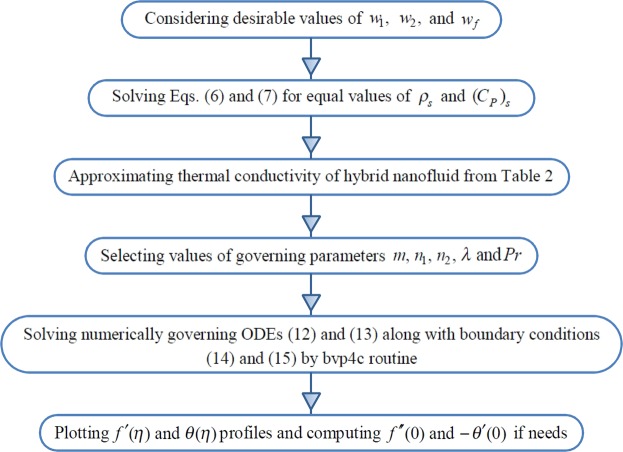
Flowchart of the present problem’s computational procedure.

Finally, after combining Eqs (11), (17) and (18), we obtain

19 $${[\frac{2R{e}_{x}}{(m+1)}]}^{\frac{1}{2}}{C}_{f}={(1-\frac{\frac{{w}_{1}+{w}_{2}}{{\rho }_{s}}}{\frac{{w}_{1}+{w}_{2}}{{\rho }_{s}}+\frac{{w}_{f}}{{\rho }_{f}}})}^{-2.5}f^{\prime\prime} (0),\,\,{[\frac{2}{(m+1)R{e}_{x}}]}^{\frac{1}{2}}N{u}_{x}=\,-\frac{{k}_{hnf}}{{k}_{f}}\theta ^{\prime} (0).$$

where $R{e}_{x}=Ux/{\upsilon }_{f}$ is the local Reynolds number. In summary, we can depict the computational procedure for our new algorithm in Fig. 2.

## Results and Discussion

The similarity governing Eqs (12) and (13) along with boundary conditions (14) and (15) are solved numerically for some values of the governing parameters *w*_1_, *w*_2_, *w*_*f*_, *ϕ*, *ϕ*_1_, *ϕ*_2_, *ρ*_*s*_, (*C*_*p*_)_*s*_, *λ*, *m*, *n*_1_, *n*_2_ and *Pr* using the bvp4c built-in function from MATLAB software (see Shampine *et al*.^55^). In this approach, we have considered $4\le {\eta }_{\infty }\le 6$, $\Delta \eta ={\eta }_{\infty }/100,$ and the relative tolerance was set as default (10^−3^). Needless to say that, we are concentrating on the real (First) solutions that have correct physical reasons.

To validate our numerical procedure, Table 4 shows the value of the similarity skin friction coefficient (*f*  ″(0)) for pure water $$(\varphi ={\varphi }_{1}={\varphi }_{2}=0),$$ static boundary $$(\lambda =0)$$ and different values of *m*. We can see from Table 4, with increasing parameter *m* the similarity skin friction coefficient enhances that it seems reasonable physically. Moreover, Table 5 shows the comparison of the values of the skin friction coefficient $${[(2R{e}_{x})/(m+1)]}^{1/2}{C}_{f}$$ and the local Nusselt number $${[2/\{(m+1)R{e}_{x}\}]}^{1/2}N{u}_{x}$$ for TiO_2_-water nanofluid with different values of $$\varphi ={\varphi }_{1}$$ and *m* while $$\lambda =0$$(stationary wedge), and $${n}_{1}={n}_{2}=3$$(Maxwell-Garnet model for $${k}_{hnf}/{k}_{f})$$. Tables 4 and 5 imply that the present results are in good agreement with previous published researches obtained by Yih *et al*.^56^, White^54^, Ishak *et al*.^40^, Yacob *et al*.^42^ and Nadeem *et al*.^44^.

**Table 4:** The values of *f*  ″(0) for various values of *m*, when $\varphi ={\varphi }_{1}={\varphi }_{2}=\lambda ={w}_{1}={w}_{2}=0,\,{w}_{f}=100\,gr$ and *Pr* = 6.2.

*m*	Yih *et al*.^56^	White^54^	Ishak *et al*.^40^	Yacob *et al*.^42^	Nadeem *et al*.^44^	Present Study
−0.0825	—	0.12864	—	—	—	0.129310
0	0.469600	—	0.4696	0.4696	0.469600	0.469600
1/11	0.654979	—	0.6550	0.6550	0.654994	0.654993
0.2	0.802125	—	0.8021	0.8021	0.802125	0.802125
1/3	0.927653	—	0.9277	0.9277	0.927680	0.927680
0.4	—	—	—	—	0.976824	0.976824
0.5	—	—	—	1.0389	1.038900	1.038903
1	1.232588	1.23259	1.2326	1.2326	1.232587	1.232587

**Table 5:** The values of ${[(2R{e}_{x})/(m+1)]}^{1/2}{C}_{f}$ and ${[2/\{(m+1)R{e}_{x}\}]}^{1/2}N{u}_{x}$ for various values of *m* and $\varphi ={\varphi }_{1}$(TiO_2_-water nanofluid) when $\lambda =0,\,{n}_{1}={n}_{2}=3$ and *Pr* = 6.2.

*m*	${\boldsymbol{\varphi }}={{\boldsymbol{\varphi }}}_{{\bf{1}}}$	${{\boldsymbol{\varphi }}}_{2}$	${[({\bf{2}}{\boldsymbol{R}}{{\boldsymbol{e}}}_{{\boldsymbol{x}}}){\boldsymbol{/}}({\boldsymbol{m}}{\boldsymbol{+}}{\bf{1}})]}^{{\bf{1}}{\boldsymbol{/}}{\bf{2}}}{{\boldsymbol{C}}}_{{\boldsymbol{f}}}$		${[{\bf{2}}{\boldsymbol{/}}\{({\boldsymbol{m}}{\boldsymbol{+}}{\bf{1}}){\boldsymbol{R}}{{\boldsymbol{e}}}_{{\boldsymbol{x}}}\}]}^{{\bf{1}}/{\bf{2}}}{\boldsymbol{N}}{{\boldsymbol{u}}}_{{\boldsymbol{x}}}$	
			Yacob *et al*.^42^	Present Study	Yacob *et al*.^42^	Present Study
0	0.1	0	0.6169	0.616929	1.0189	1.018845
	0.2	0	0.7978	0.797872	1.1561	1.156053
0.5	0.1	0	1.3648	1.364842	1.2460	1.246064
	0.2	0	1.7651	1.765142	1.4082	1.408206
1	0.1	0	1.6192	1.619291	1.3010	1.301085
	0.2	0	2.0942	2.094220	1.4691	1.469032

### Hydrodynamic and thermal boundary layers

**Figure 3:**
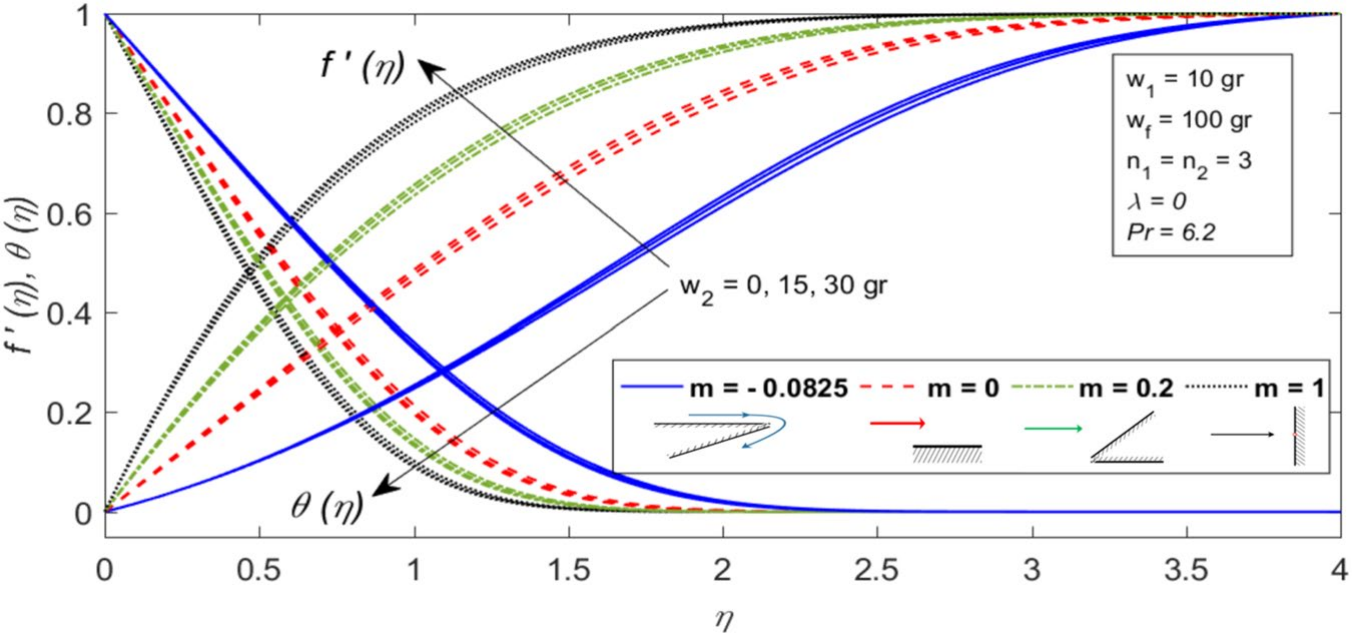
Dimensionless velocity profiles $(f^{\prime} (\eta ))$ and temperature profiles $(\theta (\eta ))$ in terms of dimensionless distance from the surface $(\eta )$ for different values of *m* and *w*_2_, when ${w}_{1}=10\,gr,\,{w}_{f}=100\,gr,\,{n}_{1}={n}_{2}=3,\,\lambda =0$ and *Pr* = 6.2.

**Figure 4:**
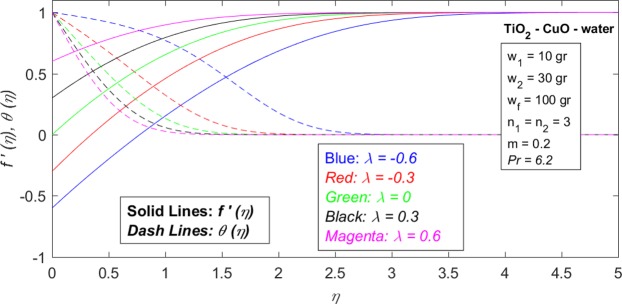
Dimensionless velocity profile $(f^{\prime} (\eta ))$ and temperature profiles $(\theta (\eta ))$ in terms of dimensionless distance from the surface $(\eta )$ for different values of *λ*, when ${w}_{1}=10\,gr,\,{w}_{2}=30\,gr,\,{w}_{f}=100\,gr,\,{n}_{1}={n}_{2}=3,\,m=0.2$ and *Pr* = 6.2.

Figure 3 shows the influence of the Falkner-Skan power law parameter (m) and mass of the second nanoparticle (*w*_2_) on the dimensionless velocity and temperature distributions, when ${w}_{1}=10\,gr,$ ${w}_{f}=100\,gr,$ ${n}_{1}={n}_{2}=3,$ $\lambda =0$ and *Pr* = 6.2. It is worth mentioning that, $m=-0.0825$ $(\beta =-0.18)$ corresponds to corner flow case before separation point, $m=0$ $(\beta =0)$ corresponds to flat plate case, $m=0.2$ $(\beta =1/3)$ corresponds to wedge flow case with $\Omega ={60}^{^\circ }$ and $m=1\,(\beta =1)$ corresponds to plane stagnation point flow case. It can be concluded that, with increasing *m* and *w*_2_ both hydrodynamic and thermal boundary layer thicknesses decrease. So, the velocity as well as the temperature gradients enhance and according to Eqs (17) and (18) the skin friction coefficient and the local Nusselt number increase. Moreover, the effect of $m$ is comparatively less in dimensionless temperature profiles at fixed ${w}_{2}$ because $m$ does not appear directly in the similarity energy Eq. (13). Figure 4 represents the aforementioned profiles for different values of *λ*, when ${w}_{1}=10\,gr,\,{w}_{2}=30\,gr,\,{w}_{f}=100\,gr,\,{n}_{1}={n}_{2}=3,\,m=0.2$ and *Pr* = 6.2. As a result of this Figure, when the wedge moves in same direction to the free stream $(\lambda  > 0)$, both hydrodynamic and thermal boundary layer thicknesses are thinner than the static wedge $(\lambda =0)$ and moving wedge in opposite direction to the free stream $(\lambda  < 0)$. So, the local Nusselt number is higher for moving wedges in same direction to the free stream $(\lambda  > 0).$

### Engineering quantities of interest: skin friction coefficient and Nusselt number

**Figure 5:**
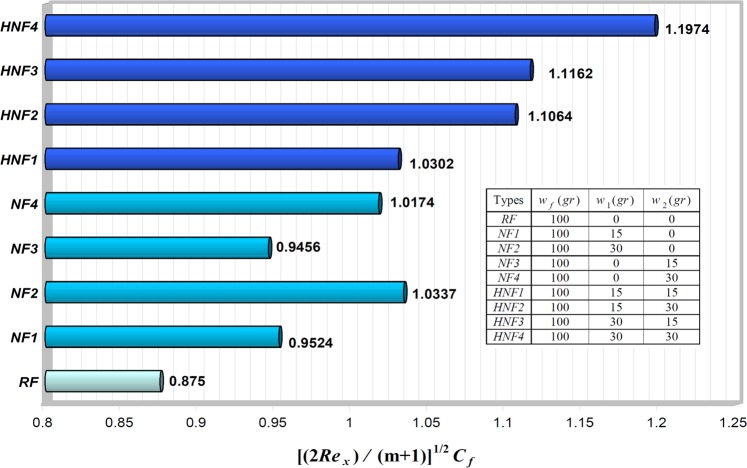
The skin friction coefficient ${[(2R{e}_{x})/(m+1)]}^{1/2}{C}_{f}$ for various values of *w*_1_ and *w*_2_, when ${w}_{f}=100\,gr,$ ${n}_{1}={n}_{2}=3,$ $\lambda =-\,0.4,$ $m=0.2$ and $Pr=6.2$.

**Figure 6:**
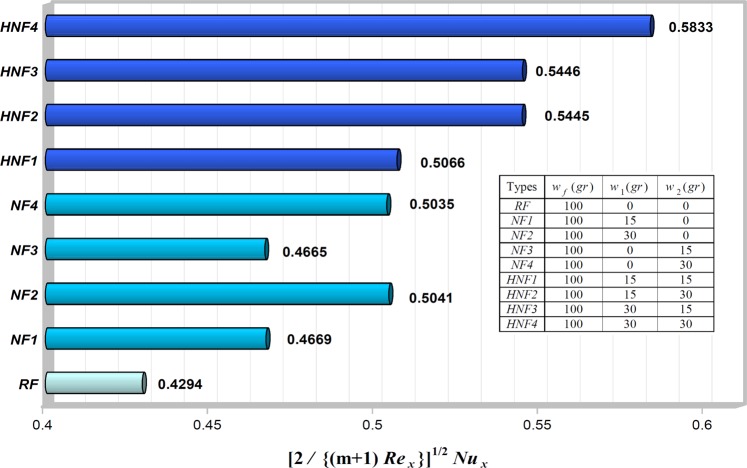
The local Nusselt number ${[2/\{(m+1)R{e}_{x}\}]}^{1/2}N{u}_{x}$ for various values of *w*_1_ and *w*_2_, when ${w}_{f}=100\,gr,$ ${n}_{1}={n}_{2}=3,$ $\lambda =-\,0.4,$ $m=0.2$ and $Pr=6.2$.

Here, we compare the skin friction coefficient ${[(2R{e}_{x})/(m+1)]}^{1/2}{C}_{f}$ and the local Nusselt number ${[2/\{(m+1)R{e}_{x}\}]}^{1/2}N{u}_{x}$ in terms of different values of first and second nanoparticle’s mass in Figs 5 and 6, respectively, by considering ${n}_{1}={n}_{2}=3,\,\lambda =-\,0.4,\,m=0.2$ and *Pr* = 6.2.

It is quite clear that, both the skin friction coefficient (the undesirable effect) and the local Nusselt number (the desired effect) increase with increasing first and second nanoparticle masses for all cases. Indeed, increasing the nanoparticles mass leads to augmenting the effective thermal conductivity, and consequently tends the heat transfer rate enhancement of our heat transfer fluid. On the other hand, according to Eq. (19), the most important factors affecting the skin friction coefficient enhancement are (i) the first and second nanoparticles as well as the base fluid masses (*w*_1_, *w*_2_ and *w*_f_) and (ii) the absolute values of the dimensionless velocity profile’s slope at the surface of the wedge (*f*  ″(0)). As a result, the skin friction coefficient enhancement always can occur by net increase of both these factors. In *HNF*4 case, we obtain the largest heat transfer rate $({[2/\{(m+1)R{e}_{x}\}]}^{1/2}N{u}_{x}=0.5833)$ and also the maximum skin friction coefficient $({[(2R{e}_{x})/(m+1)]}^{1/2}{C}_{f}=1.1974)$ between all cases that means it has better heat transfer rate relative to single nanoparticle’s nanofluid as well as pure water. So, the best status would be theoretically related to *HNF*4 case. Because in addition to having a 35% growth in heat transfer rate relative to pure water, it has a 36% increase in the skin friction coefficient. While *HNF*1, *HNF*2 and *HNF*3, respectively, have an increase in skin friction coefficient of about 17, 26, and 27%, compared to the base fluid. Nevertheless, checking the optimal range for these mass-based cases will require further field studies in the future. However, our major challenge is the high skin friction that requires the high pressure drop and the high relevant pumping power. Therefore, we always should control this issue for practical applications. After all, we can deduce that hybrid nanofluids sufficiently can be used in all applications where ever single nanoparticle’s nanofluids have been used.

### Influence of nanoparticles shape on thermal characteristics of problem

**Figure 7:**
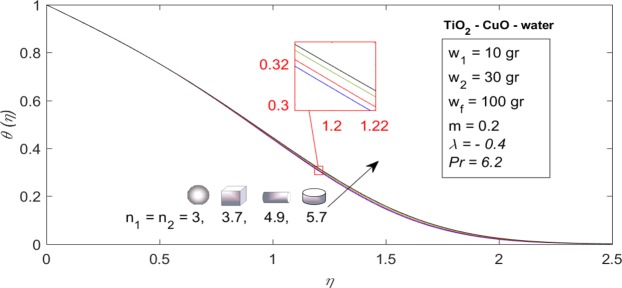
Dimensionless temperature profiles $(\theta (\eta ))$ for some values of ${n}_{1}={n}_{2}$ when ${w}_{1}=10\,gr,$ ${w}_{2}=30\,gr,$ ${w}_{f}=100\,gr,$ $\lambda =-0.4,$ $m=0.2$ and *Pr* = 6.2.

**Figure 8:**
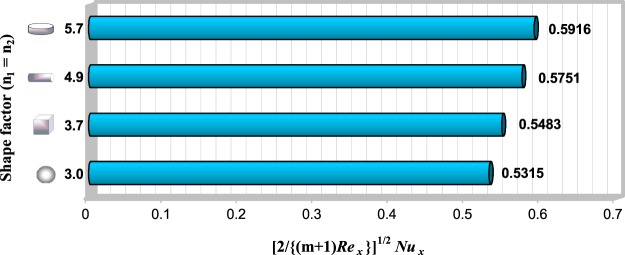
The local Nusselt number ${[2/\{(m+1)R{e}_{x}\}]}^{1/2}N{u}_{x}$ for some values of ${n}_{1}={n}_{2}$, when ${w}_{1}=10\,gr,$ ${w}_{2}=30\,gr,$ ${w}_{f}=100\,gr,$ $\lambda =-0.4,$ $m=0.2$ and *Pr* = 6.2.

Figure 7 demonstrates dimensionless temperature profiles for some values of nanoparticle’s shape factor $({n}_{1}={n}_{2})$ that were exhibited in Table 3, when ${w}_{1}=10\,gr,\,{w}_{2}=30\,gr,\,{w}_{f}=100\,gr,\,\lambda =-\,0.4,\,m=0.2$ and $Pr=\mathrm{6.2.}$ As we know, the nanoparticles shape factor (*n*_1_ and *n*_2_) only affect the thermal characteristics of the problem due to their representations in the similarity energy Eq. (13) (see Eq. (13) and the thermal conductivity approximation of hybrid nanofluid in Table 2). On the other hand, values of local Nusselt number of nanoparticles shape factor from Fig. 7 are depicted in the bar diagram of Fig. 8. It is worth mentioning that, Fig. 8 illustrates when the nanoparticles shape is platelet $({n}_{1}={n}_{2}=5.7)$, we possess largest heat transfer rate $({[2/\{(m+1)R{e}_{x}\}]}^{1/2}N{u}_{x}=0.5916)$ while, the opposite trend is valid for spherical shape of nanoparticles $({n}_{1}={n}_{2}=3)$.

Finally, in Table 6 we have compared the local Nusselt number of different shapes of first (TiO_2_) and second (CuO) nanoparticles (*n*_1_ and *n*_2_) in terms of different hybrid nanofluid masses that are tabulated in Figs 5 and 6 (entitled *HNF*1-*HNF*4), when ${w}_{f}=100\,gr,\,\lambda =-\,0.4,\,m=0.2$ and $Pr=6.2$. It is seen that the local Nusselt number enhances with elevating shape factor of first or second nanoparticles in all cases. Further, it is perceived that, generally when the shape of second nanoparticle is spherical $({n}_{2}=3)$ while, the shape of first nanoparticle is not spherical $({n}_{1}\ne 3),$ the heat transfer rate of hybrid nanofluid is higher relative to opposite ones.

**Table 6:** The local heat transfer rate $({[2/\{(m+1)R{e}_{x}\}]}^{1/2}N{u}_{x})$ for some values of *n*_1_ and *n*_2_ based on various cases of hybrid nanofluids mass, when $\lambda =-\,0.4,\,m=0.2$ and $Pr=6.2$.

Types	${{\boldsymbol{n}}}_{{\bf{1}}}{\boldsymbol{=}}{\bf{3}}$				${{\boldsymbol{n}}}_{{\bf{2}}}{\boldsymbol{=}}{\bf{3}}$			
	${{\boldsymbol{n}}}_{{\bf{2}}}{\boldsymbol{=}}{\bf{3}}$	${{\boldsymbol{n}}}_{{\bf{2}}}{\boldsymbol{=}}{\bf{3.7}}$	${{\boldsymbol{n}}}_{{\bf{2}}}{\boldsymbol{=}}{\bf{4.9}}$	${{\boldsymbol{n}}}_{{\bf{2}}}{\boldsymbol{=}}{\bf{5.7}}$	${{\boldsymbol{n}}}_{{\bf{1}}}{\boldsymbol{=}}{\bf{3}}$	${{\boldsymbol{n}}}_{{\bf{1}}}{\boldsymbol{=}}{\bf{3.7}}$	${{\boldsymbol{n}}}_{{\bf{1}}}{\boldsymbol{=}}{\bf{4.9}}$	${{\boldsymbol{n}}}_{{\bf{1}}}{\boldsymbol{=}}{\bf{5.7}}$
*HNF1*	0.5066	0.5125	0.5218	0.5276	0.5066	0.5137	0.5244	0.5308
*HNF2*	0.5445	0.5565	0.5757	0.5876	0.5445	0.5520	0.5634	0.5702
*HNF3*	0.5446	0.5508	0.5605	0.5666	0.5446	0.5589	0.5805	0.5933
*HNF4*	0.5833	0.5959	0.6159	0.6282	0.5833	0.5984	0.6214	0.6349

## Conclusions

The laminar two-dimensional Falkner-Skan problem by taking Newtonian TiO_2_-CuO/water hybrid nanofluid into account as the working liquid and with constant surface temperature was investigated semi-analytically with help of new proposed algorithm according to nanoparticles and base fluid masses. Our hypothesize was that the Prandtl number of water is 6.2. After implementing Tiwari-Das single-phase nanofluid model, non-dimensional form of the governing PDEs were written using auxiliary similarity variables, then we attempted to numerically solve them by bvp4c function from MATLAB. The major conclusions of this research, may be summarized as follows: (1) the Falkner-Skan power law parameter (*m*) and the second nanoparticle mass (*w*_2_) increase the local Nusselt number at the surface of the wedge, (2) the local Nusselt number is higher for moving wedges in same direction to the free stream $(\lambda  > 0)$ relative to static wedges $(\lambda =0)$ as well as moving wedges in opposite direction to the free stream $(\lambda  < 0)$, (3) mass increment of first and second nanoparticles invoke enhancement on skin friction and local heat transfer rate of our hybrid nanofluid, (4) when the nanoparticle shape is spheric, the local Nusselt number will be minimum than other nanoparticle shapes, (5) the *HNF*4 case with highest nanoparticles mass, possesses the largest local heat transfer rate between other mass-based cases, that means it has better thermal performance relative to mono-nanofluid and base fluid, respectively.
